# Profiling of Junior College Football Players and Differences between Position Groups

**DOI:** 10.3390/sports4030041

**Published:** 2016-08-05

**Authors:** Robert G. Lockie, Adrina Lazar, Ashley J. Orjalo, DeShaun L. Davis, Matthew R. Moreno, Fabrice G. Risso, Matthew E. Hank, Randal C. Stone, Nicholas W. Mosich

**Affiliations:** 1 Department of Kinesiology, California State University, Fullerton, CA 92834, USA; 2 Department of Kinesiology, California State University, Northridge, CA 91330, USA; adrina.lazar.957@my.csun.edu (A.L.); ashley.orjalo.793@my.csun.edu (A.J.O.); deshaun.davis.907@my.csun.edu (D.L.D.); matthew.moreno.595@my.csun.edu (M.R.M.); fabrice.risso.893@my.csun.edu (F.G.R.); randal.stone.456@my.csun.edu (R.C.S.); nicholas.mosich.685@my.csun.edu (N.W.M.); 3 Department of Athletics, Santa Monica College, Santa Monica, CA 90405, USA; hank_matthew@smc.edu

**Keywords:** American football, community college, body mass, linear and change-of-direction speed, lower-body power

## Abstract

This study profiled junior college football players. Sixty-two subjects completed vertical jump (VJ; height and peak power), standing broad jump (SBJ), 36.58 m sprint, pro-agility shuttle, three-cone drill, and maximal-repetition bench press and front squat. The sample included 2 quarterbacks (QB), 7 running backs (RB), 13 wide receivers (WR), 1 tight end (TE), 18 defensive backs (DB), 8 linebackers (LB), and 13 offensive and defensive linemen (LM). To investigate positional differences, subjects were split into skill (SK; WR, DB), big skill (BSK; QB, RB, TE, LB), and LM groups. A one-way ANOVA determined between-group differences. LM were taller and heavier than SK and BSK players. The SK and BSK groups were faster than LM in the 0–36.58 m sprint, pro-agility shuttle, and three-cone drill (*p* ≤ 0.009). The SK group had greater VJ height and SBJ distance; LM generated greater VJ peak power (*p* ≤ 0.022). There were no between-group differences in the strength endurance tests. Compared to Division I data, junior college players were smaller, slower, and performed worse in jump tests. Positional differences in junior college football are typical to that of established research. Junior college players should attempt to increase body mass, and improve speed and lower-body power.

## 1. Introduction

College athletics in America, which is administered by the National Collegiate Athletic Association (NCAA), is a multi-billion-dollar industry, with football being the most dominant sport [1]. The highest level of collegiate competition is Division I (followed by Divisions II and III), and there is great competition between schools with the recruitment of athletes to their respective programs. Recruiting the best players possible is incumbent on those who run athletic programs, and Division I and II schools can offer scholarships to entice athletes to their programs [2]. High-quality recruits to a football program can have a major impact for a university, not only on the field [3], but financially as well [4]. Traditionally, most recruits to a football program will come from high schools. However, acquiring junior (or community) college transfers is another method by which players can be brought into a program [5].

Junior college athletes will generally spend two years within a program at this level, before transferring to a four-year institution where they will have academic credit and the opportunity to participate in athletics. As high school football players will often need time to physically mature and develop before they are competitive with experienced collegiate players [6,7], the same could be expected of junior college players [5]. Indeed, more experienced athletes within a collegiate program will be more physically developed when compared to their younger counterparts [5,8,9]. This may mean that a player who is lacking certain qualities may not be able to physically compete against their opponents, or even their teammates during practice. For example, Carbuhn et al. [5] found body mass discrepancies of approximately 6–8 kilograms (kg) between junior college transfers and established Division I quarterbacks (QB), running backs (RB), and defensive linemen. Junior college linemen who have transferred into a Division I program were found to be approximately 10 kg lighter than their established Division I counterparts, while linebackers (LB) were almost 20 kg lighter [5]. A junior college transfer, who is notably lighter than the established players from the program, will have difficulty in competing during practice, which could then influence their ability to earn playing time.

A further issue is that there has been little research of the specific athletic population of junior college football players [5,10,11], and some of the research that does exist is over 20 years old [10,11]. Additionally, there has been no direct documentation of many of the important physiological characteristics of junior college football players. Carbuhn et al. [5] measured maximal strength of junior college transfers into a Division I program, including one-repetition maximum (1RM) bench press, squat and power clean, as well as the vertical jump (VJ). Across almost every position, Carbuhn et al. [5] found that junior college transfers exhibited lower levels of strength, and power as defined by VJ height. However, Carbuhn et al. [5] did not measure characteristics such as linear and change-of-direction (COD) speed, despite their importance to football [12,13,14,15]. It would be valuable to measure the performance of junior college football players in tests common to the sport—such as the 36.58 meter (m) (40 yard) sprint, pro-agility shuttle, and three cone-drill—which has not yet been documented in the scientific literature.

Dos Remedios and Holland [11] conducted a survey of 35 junior college football programs, to compare the data to the Division I football players profiled by Berg et al. [16]. The data indicated that junior college players were generally smaller in stature, weaker in maximum bench press and squat strength tests, and slower in the 36.58 m sprint. This is notable, given the importance of qualities such as body size, strength, power, and speed for football [9,12,13,15,17,18,19]. It would be beneficial to collegiate strength and conditioning coaches to know the traits of junior college players before they enter higher-level college football competition. Additionally, there is also value in determining whether the positional characteristics of junior college players is similar to that established for high school [6,13,20], collegiate [16,21,22,23,24,25], and professional [12,26,27] players. Establishing a baseline of performance assessments for a sample of junior college players will provide useful information for football and strength and conditioning coaches, in that it will elicit greater understanding of the characteristics of players who may enter their program from this level of competition. Although coaches will always want to directly assess the athletic performance of potential junior college recruits to their program, access to data of a sample of equivalent players will provide context for any measurements that are collected.

Therefore, this study documented the characteristics of football players from a junior college that has regularly had players transfer to Division I programs. Although this sample draws from only one school, this research does provide a preliminary, detailed analysis of a specific sample of football players that has been under-investigated in the literature. The players were assessed in stature and body mass, jump performance, linear and COD speed, and strength endurance [5,6,12,13,18,20,21,22,23,24,28]. The subjects were also split into skill (SK), big skill (BSK), and linemen (LM) groups to compare the physical and physiological traits across positions. A hypothesis was made that the SK and BSK groups would perform better in the performance tests, but the LM would be physically taller and heavier. Furthermore, it was hypothesized that when compared to data from Division I and II players, the junior college players would generally be smaller in stature, and perform poorer in the performance tests. As stated, determining a performance baseline for junior college football players will provide useful information for coaches as they will be more aware of the characteristics of players from this competition level. Furthermore, junior college players will also recognize those physiological characteristics they need to improve in order to be competitive at higher levels of play.

## 2. Materials and Methods

### 2.1. Subjects

Sixty-two junior college football players (age = 20.11 ± 1.60 years; height = 1.83 ± 0.07 m; mass = 93.66 ± 14.16 kg) from one school were recruited for this study. By position, there were 2 QB, 7 RB, 13 wide receivers (WR), 1 tight end (TE), 18 defensive backs (DB), 8 LB, and 13 offensive (centers, guards, and tackles) and defensive (defensive tackles and ends) LM. Due to the relatively small number of LM tested compared to other positions, as well as the fact that at the junior college level LM will often play offense and defense, all LM were grouped together [20]. Nonetheless, the sample included all members of the squad that were currently participating in full training. Subjects were further split into SK (WR and DB), BSK (QB, RB, TE, and LB), and LM (offensive centers, guards, and tackles; defensive tackles and ends) groups [9,12], in order to compare the position groups. The data utilized for this study were collected as a condition of player monitoring in which activities are routinely measured over the course of the season by the team’s strength and conditioning staff [29]. As a result, the institutional ethics committee approved the use and analysis of preexisting data. The research still conformed to the recommendations of the Declaration of Helsinki, and all subjects received a clear explanation of the study, including the risks and benefits of participation. All subjects had also completed the appropriate screening and consent forms in order to participate in collegiate athletics.

### 2.2. Procedures

Data was collected during the months of April and May, which was the preseason period for the team. All subjects were familiar with the tests performed in this study, as they were consistently used by the team’s strength and conditioning staff for general player monitoring. Jump and strength assessments were completed over three sessions; the VJ in session one, the standing broad jump (SBJ) and front squat in session two, and the bench press in the last gym session. Speed assessments were completed across three field sessions following the team’s usual warm-up. These incorporated the: (1) 36.58 m sprint; (2) pro-agility shuttle; and (3) three-cone drill. Each testing session lasted for approximately 60 min in duration. Testing was conducted to fit into the schedule designed by the team’s head coach and strength and conditioning staff, and at least 48 h was provided between each testing session. In the gym-based session, subjects wore their own athletic trainers, and testing was conducted on a rubber-matted floor. Field testing was conducted on a grass outdoor pitch; subjects wore their own cleats.

Prior to data collection in the first gym session, the subject’s age, height, and body mass were recorded. Height was measured barefoot using a portable stadiometer (Seca 213, Ecomed Trading, Australia). Body mass was recorded using electronic digital scales (BF-522, Tanita Corporation, Japan). The gym-based session was preceded by a standardized warm-up designed by the team’s coaching staff, consisting of 10 min of jogging, and 10 min of dynamic stretching. Subjects also completed a standardized warm-up before each field session that was designed by the team’s coaching staff, which consisted of 10 min of jogging, 10 min of dynamic stretching, and linear and lateral runs over 20–30 m that progressively increased in intensity. Subjects completed each test by rotating alphabetically by surname within each session [18,20]. This was done to ensure sufficient recovery periods (greater than 3 min) between efforts for each subject. A standard metric tape measure was used to determine all distances.

### 2.3. Vertical Jump (VJ)

The VJ was used to indirectly measure leg power in the vertical plane [18,30]. A Vertec apparatus (JUMPUSA, Sunnyvale, CA, USA) was used to measure jump height. Assessing VJ using the Vertec has been found to have high reliability in active males (intra-class correlation coefficients (ICC) = 0.94; coefficient of variation (CV) = 4.6%–5.5%) [31]. The subject initially stood side-on to the Vertec (on the subjects’ dominant side), and while keeping their heels on the floor, reached upward as high as possible, fully elevating the shoulder to displace as many vanes as possible. The last vane moved became the zero reference. The subject then jumped as high as possible with no preparatory step, and height was recorded from highest vane moved and converted to a measurement in meters. No restrictions were placed on the knee angle attained during the eccentric phase of the jump. VJ height was calculated by subtracting the standing reach height from the jump height. Each subject completed two trials, and the best trial was used for analysis. Peak power from the VJ was also calculated for the best trial by using the formula from Johnson and Bahamonde [32]: Peak Power (watts) = (78.6 VJ height) + (60.3 body mass) − (15.3 body height) − 1245. Power was measured in watts (w).

### 2.4. Standing Broad Jump (SBJ)

The SBJ was used to indirectly measure leg power in the horizontal plane. This test was performed according to established methods [13,18,20], and is reliable (ICC = 0.95; CV = 2.4%) [33]. The subject placed the toes of both feet on the back of the starting line, and with a simultaneous arm swing and crouch, leapt as far forward as possible, ensuring a two-footed landing. Subjects had to execute a correct landing for the trial to be counted; if not, the trial was disregarded and reattempted. No restrictions were placed on range of countermovement during the preparatory phase of the jump, or the arm swing used. Distance was measured using a standard tape measure, which was the perpendicular line from the front of the start line to the posterior surface of the back heel at the landing. Each subject completed two trials, and the best trial was used. The SBJ distance for the best trial was made ratio scaled relative to body mass via the formula: relative SBJ (m·kg^−1^) = jump distance (m) ÷ body mass (kg) [18].

### 2.5. Strength Endurance Testing—Bench Press and Front Squat

The strength endurance testing in this research involved the bench press for the upper-body, and the front squat for the lower-body. The coaching staff for this football squad only tested strength endurance as opposed to maximal strength in their athletes, due to a perceived risk of injury with RM strength testing [34,35,36]. Both tests were adapted from the typical combine strength test of the 102 kg (225 pounds) bench press, whereby subjects attempt to complete as many repetitions as possible [27,28,37,38,39]. The 102 kg bench press test has been found to have excellent reliability (ICC = 0.98–0.99; CV = 7.7%–8.9%) [40]. Furthermore, following a review of literature, Pereira and Gomes [41] documented strength endurance testing using exercises such as the bench press and squat, which has had good reliability (ICC > 0.80). A standard Olympic bar and weight plates were used for both the bench press and front squat. When performing the bench press, subjects lay supine on a flat bench with their feet flat on the floor, and their head, shoulders and buttocks flat to the bench. Subjects used a pronated, slightly wider than shoulder-width grip on the bar, and completed a personal preference warm-up using 50%–75% of their estimated 1RM [38]. This was followed by a rest period of at least 3 min, before subjects then completed as many repetitions as possible at their assigned load. The WR and DB (i.e., the SK group) lifted a bench press load of 84 kg (185 pounds) for as many repetitions as possible. All other positions (i.e., the BSK and LM groups) lifted a bench press load of 102 kg (225 pounds) for as many repetitions as possible. These different loads have been used previously to assess football players with lower (84 kg/185 pounds) and higher (102 kg/225 pounds) levels of strength [28]. The ‘touch-and-go’ procedure was adopted, in that the bar was required to touch the chest before being pressed to full arm extension for each repetition. A repetition was deemed to be successful when the bar was moved from the chest to a position of full elbow extension. Failure to do this, or bouncing the bar on the chest, raising the feet, or the buttocks leaving the bench, disqualified a repetition [39]. No time limitations were placed on this test; the test continued until the subject could no longer move the bar [39]. The researchers counted the number of successful repetitions performed during the bench press.

Although the back squat has been used for strength testing in football players [5,22,25], the junior college’s strength and conditioning staff chose to use the front squat over the back squat for safety reasons with relatively younger lifters [42]. As stated by Gullett et al. [42], the front squat results in lower compressive forces in the tibiofemoral joint, while still producing similar patterns of lower-body muscle activity when compared to the back squat. The use of the front squat in this study limits the comparisons that can be made with established research in the scientific literature. Nevertheless, the between-position analyses using strength endurance data measured by the front squat are still relevant in the context of this research. Similar to the bench press warm-up, subjects completed a personal preference warm-up with 50%–75% of perceived 1RM [38], rested 3 min, and then completed the front squat with their assigned load. The WR and DB squatted a load of 84 kg for as many repetitions as possible, while all other positions squatted a load of 102 kg [28]. Subjects were instructed to descend until the tops of their thighs were parallel to the floor before attempting to ascend. This was visually assessed by the researchers, and subjects were also given verbal cues on when they were to halt the down phase, and begin the up phase, of the front squat. The pins were adjusted in the rack and placed as close as possible to the bottom of the final position of the bar. No time limitations were placed on the front squat test; the test continued until the subject could no longer move the bar [39]. The researchers counted the number of successful repetitions performed. To allow for further between-group comparisons, tonnage for the bench press and front squat was calculated by multiplying the load by the number of completed repetitions for both lifts [43]. In addition to the absolute value, tonnage was also ratio-scaled relative to body mass (relative tonnage (kg·BM^−1^) = tonnage∙body mass^−1^), and allometrically scaled [44,45,46]. The allometric scaling for bench press tonnage was derived via the formula of tonnage∙body mass^−0.67^ [44,45,46]. The allometric scaling for front squat tonnage was calculated via tonnage∙body mass^−0.33^ [44,46].

### 2.6. 36.58 Meter (m) Sprint

The 36.58 m sprint was used to measure linear speed specific to football [12,13,18,20,26,27,37], and sprint time was recorded by a timing lights system (Smartspeed, Fusion Sports, Sumner Park, Australia). The reliability of using this system in sprint testing has been documented (ICC = 0.76–0.96; CV = 1.9%–5.1%) [47]. Gates were positioned at 0 m, 4.57 m (5 yards), 9.14 m (10 yards), and 36.58 m (40 yards) to measure the 0–4.57 m, 0–9.14 m, and 0–36.58 m intervals. The 0–4.57 m was measured due to the importance of fast initial steps during acceleration in field sport athletes [30]. The 0–9.14 m interval is often measured during the 36.58 m sprint to assess acceleration [18,20,23,27,37]. Subjects began the sprint from a three-point stance 50 cm behind the start line, to be able to trigger the first gate. Subjects were instructed to accelerate from the starting line and sprint through all sets of timing lights. If the subjects moved prior to starting, the trial was disregarded and repeated. Subjects completed two trials, and the fastest trial was used for analysis. Time for each distance was recorded to the nearest 0.001 seconds (s).

### 2.7. Pro-Agility Shuttle

**Figure 1:**
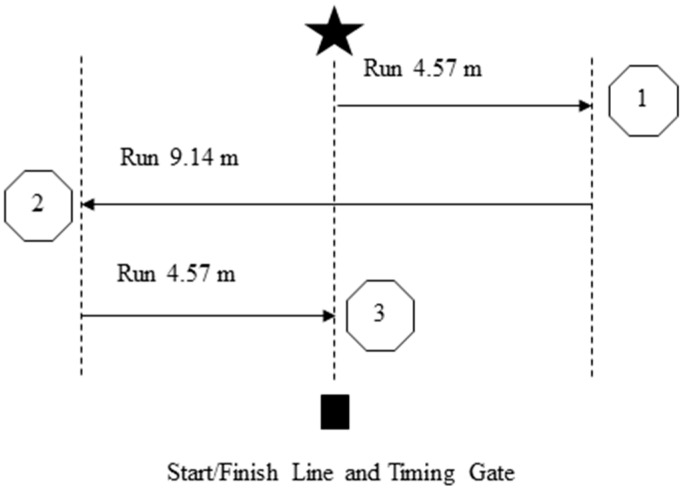
Pro-agility shuttle test with initial movement to the right. m = meters.

The pro-agility shuttle course and running path is shown in Figure 1, and the test was completed as per established methods [6,9,12,18,20]. Further, this test has been shown to be reliable in male and female team sport athletes (ICC = 0.90; CV = 2.19%) [48]. One timing gate (Smartspeed, Fusion Sports, Sumner Park, Australia) was used for this test, set at a height of approximately 1 m. Subjects straddled the middle line in a three-point stance in between the timing gate. An in-beam start was used, whereby once the subject was stable in the light beam, they could begin the test [18,20]. In order to initiate the test, the subject turned and ran 4.57 m (5 yards) to one side and touched the line with one hand. The subject then turned and ran 9.14 m (10 yards) to the other side and touched the other line, before turning and finishing by running back through the start/finish line. Observers were positioned at either end of the pro-agility shuttle to ensure subjects touched the line; if they did not, the trial was disregarded and reattempted. The timing system started when the subject left the light beam, and stopped recording when subjects returned through the gate for the last time. Two trials were completed—one with movement initiation to the left, and the other with movement initiation to the right [6,12,18,20]. The fastest time from the two trials was utilized. Time for each trial was recorded to the nearest 0.001 s.

### 2.8. Three-Cone Drill

**Figure 2:**
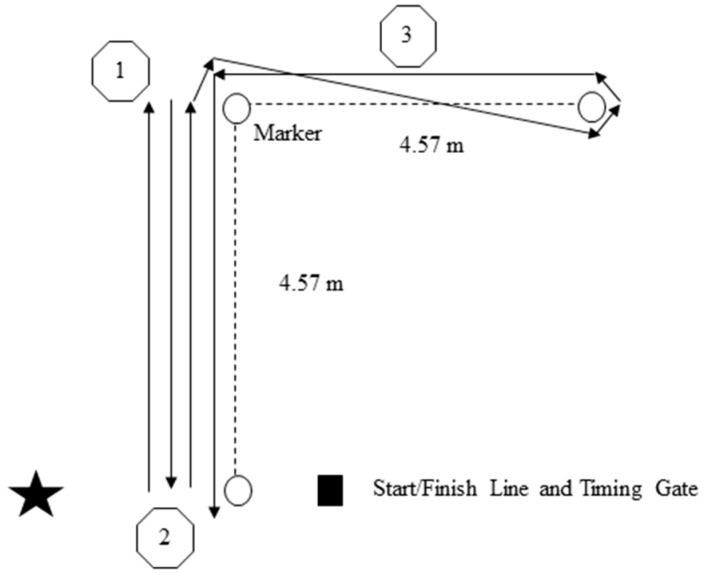
The three-cone drill featuring a right-hand turn. m = meters.

The three-cone drill was marked out as shown in Figure 2 [12,18,20], and has also been found to be reliable in male and female team sport athletes (ICC = 0.94; CV = 1.96%) [48]. One timing gate (Smartspeed, Fusion Sports, Sumner Park, Australia) was used for this test. Subjects started in a three-point stance 50 cm behind the start line (Marker 1), so as to be able to trigger the gate. After starting, subjects ran to Marker 2, bent down and touched the ground, before running back to Marker 1 and touching the ground again. The subject then ran back to Marker 2 and around the outside of it, weaved inside Marker 3, around the outside of Markers 3 and 2 before finishing at Marker 1. The subject ran forward throughout the test while altering their running direction. Observers were positioned at each marker to ensure subjects completed the requirements of the three-cone drill. If they did not, the trial was disregarded and reattempted. Time was recorded from when the subject broke the gate the first time, until they returned through the gate from the last section of the test. Two trials were completed. In the first trial, subjects turned to the right from Markers 2 to 3. In the second trial, subjects turned to the left [12,18,20]. The fastest time from the two trials was used for analysis, and time for each trial was recorded to the nearest 0.001 s.

### 2.9. Statistical Analysis

All statistical analyses were processed using the Statistics Package for Social Sciences (Version 22.0; IBM Corporation, New York, NY, USA). Descriptive data (means ± standard deviations (SD); 95% confidence intervals (CI)) were calculated by positions (QB, RB, WR, TE, LB, DB, and LM), so that the data can be presented as an initial normative profile and compared to existing data from Divisions I, II, and III collegiate football players. The Shapiro–Wilk test was conducted to ensure a normal distribution of data for each variable. Alpha levels ranged from *p* = 0.124–1.000, and thus parametric statistical calculations were used in this study. As previously acknowledged, to further investigate positional characteristics, subjects were grouped together as SK (WR and DB), BSK (QB, RB, TE, and LB), and LM [9,12]. A one-way analysis of variance, with Bonferroni post hoc for multiple pairwise comparisons, was used to calculate any differences between the SK, BSK, and LM groups. Statistical significance was set at *p* < 0.05. Effect sizes (*d*) were also calculated for the pairwise comparison, where the difference between the means was divided by the pooled SD [49]. In accordance with Hopkins [50], a *d* less than 0.2 was considered a trivial effect, 0.2–0.6 a small effect, 0.6–1.2 a moderate effect, 1.2–2.0 a large effect, 2.0–4.0 a very large effect, and 4.0 and above an extremely large effect.

## 3. Results

Table 1 displays the data for age, height, and body mass for the individual positions, and the position groups. There were no differences in age between the SK, BSK, and LM groups. LM were significantly taller, with a moderate effect, than both the SK and BSK groups. The LM were also significantly heavier than the SK (very large effect) and BSK groups (moderate effect), and the BSK players were heavier than the SK players (moderate effect).

Table 2 displays the data for the speed tests. Certain position players were absent on select testing days which meant data was not recorded for the QB in the pro-agility shuttle, or for the TE in the three-cone drill. There were no significant between-group differences in 0–4.57 m time. The SK players were 7% faster than LM in the 0–9.14 m interval (large effect). There was a moderate effect for the time difference between the BSK and LM, but it was not significant. When compared to LM, the SK and BSK groups were both 9% significantly faster in the 0–36.58 m sprint interval (moderate effects), and 8% faster in both the pro-agility shuttle (large effects) and three-cone drill (very large effects).

Jump data is shown in Table 3. The QB were absent on the VJ testing day. The SK group had a greater VJ height when compared to the LM (large effect). There was no significant difference in VJ height when comparing the BSK and LM, although there was a moderate effect. LM generated significantly greater VJ peak power compared to the SK (very large effect) and BSK groups (moderate effect). The BSK also had a greater VJ peak power compared to the SK group (large effect). The SK group had a significantly greater SBJ distance and relative SBJ when compared to the LM (large and very large effects, respectively), and greater relative SBJ when compared to the BSK group (large effect). The BSK group had a greater relative SBJ compared to the LM (large effect).

One QB was unable to complete the strength endurance tests. The data for the bench press are shown in Table 4. There were no significant differences in number of repetitions completed, tonnage, or ratio-scaled or allometrically scaled tonnage. There were also no significant between-group differences for any of these variables for the front squat (Table 5). There were moderate effects for the greater number of repetitions completed, as well as the greater ratio-scaled and allometrically scaled tonnage, for the SK and BK front squats compared to the LM. However, as stated, these differences were not significant.

## 4. Discussion

This is the first study to provide a detailed profile of junior college American football players. Previous research has detailed the characteristics of Divisions I [5,16,21,22,25], II [21], and III [23,24] football players, such that certain results from this study can be contextualized. In addition to this, players were split into SK, BSK, and LM groups to ascertain whether junior college players exhibited the same characteristics as that established for football players in the literature. Although this study included data from players from only one school, this research still provides a preliminary analysis of a specific sample of junior college football players, who have been under-investigated in the literature. Furthermore, while it should be acknowledged that there are limitations as to the predictive capabilities of testing data [26,37], this data still provides an understanding of those characteristics that have been deemed important to this sport [15]. Regarding the performance tests used in this study, it was hypothesized that the SK and BSK players would perform better when compared to LM. Generally, this hypothesis was confirmed. A further hypothesis was that higher-level players from Division I school would be physically bigger, and perform better in the football-specific tests. As will be discussed, to an extent this was confirmed as well.

Collegiate football players have generally increased in both height and body mass over the past few decades, and this has been especially pronounced for LM [19]. The LM in this study were taller and heavier than both the BSK and SK groups, which was expected, while the BSK group was also heavier than the SK players (Table 1). The nature of the position for LM dictates the need for greater body size, and these results are reflective of the literature [12,13,15,19,20,21,22]. This is also true for the different players from the BSK group (i.e., QB, RB, TE, and LB), as players from these positions play closer to the line of scrimmage. As a result, these players will be more involved with the collisions inherent to football [15,51]. The junior college players from this study tended to be larger in stature across the different positions than Division III players that were investigated by Stuempfle et al. [23]. Division III players do not receive athletic scholarships to play football, which would suggest that they are not the type of players to be recruited to more prominent Division I or II schools. Most positions were also comparable in height to Division I players, and the WR, TE, DB, and LB were similar in body mass compared to this population [21,22]. There were, however, differences in body mass for certain positions. The two QB from this study were almost 10 kg lighter than the QB from Secora et al. [22], while the RB were approximately 5 kg lighter than those from Garstecki et al. [21]. The LM from this study were between 10 and 20 kg lighter than Division I LM [21,22], which is a greater difference than that shown by Carbuhn et al. [5]. Junior college players who wish to progress to Division I or II may need to increase body mass to be physically competitive. However, coaches should determine this with individual players they wish to recruit.

Due to the structure of the game, football heavily stresses a player’s anaerobic capacities, in particular linear and COD speed [14,15]. Interestingly, there were no significant differences in the 0–4.57 m sprint interval (Table 2). This could relate to the high-intensity, acceleration demands placed upon all football positions due to the structure of the sport [15]. In contrast to this, the SK players were faster than LM over the 0–9.14 m interval, and both the SK and BSK players were faster than LM over the 0–36.58 m interval. This was expected, as the players featured in these positon groups (e.g., RB, WR, and DB) are commonly the fastest players on a football team [12,13,15,20,21,22]. Additionally, the SK and BSK players have a heavy requirement for COD actions during match-play [15]. Similar to previous research [6,13,20,22], the SK and BSK groups were faster in the tests of COD speed; the pro-agility shuttle and three-cone drill (Table 2).

The findings from this study also highlighted the limitations in the linear and COD speed capacities of junior college football players. Although the subjects from this study were generally faster in the 36.58 m sprint and pro-agility shuttle in comparison to Division III football players [23], when compared to Division I and II players assessed by Garstecki et al. [21], the junior college players were slower across all positions. This was also the case when considering football players at the end of their collegiate careers who were entering the National Football League [12]. Although Sierer et al. [12] used slightly different position groups when compared to the current study (i.e., RB were placed in the SK group, defensive ends were in the BSK group, and QB were not analyzed), the junior college players from this research (data shown in Table 2) were notably slower in the 36.58 m sprint (SK = ~4.542 s; BSK = ~4.787 s; LM = 5.257 s), pro-agility shuttle (SK = ~4.147 s; BSK = ~4.279 s; LM = 4.661 s), and three-cone drill (SK = ~7.116 s; BSK = ~7.254 s; LM = 7.855 s). The sample of junior college players from this study appeared to have limitations in linear and COD speed. Although improving speed in athletes can be challenging, recent meta-analyses and reviews of the literature have indicated that appropriate strength and sprint training can lead to improvements in running speed in trained individuals [52,53]. Ideally, to help prepare for the next level of collegiate play, junior college football players should attempt to improve all aspects of speed, including acceleration, maximal velocity, and COD speed. A range of modalities (e.g., free, assisted, and resisted sprinting; plyometrics; strength training) can be used to enhance running speed [52,54], so coaches should adopt these in the developmental training of junior college players. The coach should assess linear and COD speed for those individual players that are being recruited to their respective program, and use this data to target any shortcomings that may exist.

The SK group performed best in the VJ, SBJ, and relative SBJ (Table 3). This is typical for WR and DB, who often exhibit high lower-body power measured by jump tests [13,20,21,22]. This is likely due to the nature of the WR and DB positions, where players will need to have a good VJ to contest the ball when it is in the air. However, the LM, possibly due to their greater body mass, generated a higher VJ peak power than the BSK and SK groups (Table 3). In line with this, the BSK group also generated greater peak power than the SK group (Table 3). The football players from this study were comparable in the VJ when compared to Division II players [21], and superior when compared to Division III players [23]. As for the speed tests, the Division I football players assessed by Garstecki et al. [21] were superior across all positions in the VJ when compared to the junior college players from this research. This was also true when comparing the current studies’ subjects with the player groups analyzed by Sierer et al. [12], who possessed VJ performances that were approximately 0.2 m superior to the SK, BSK, and LM groups from this research. In addition to this, the players analyzed by Sierer et al. [12] were superior by approximately 0.3 m in the SBJ. Although this recommendation should be contextualized with the one school sample from this study, junior college football players should attempt to improve lower-body power when attempting to progress to Division I competition. Plyometrics is one training modality that should be emphasized in junior college players to enhance their lower-body power [55,56]. Coaches should also ascertain the lower-body power capabilities on a recruit-by-recruit basis. Given that lower-body power serves as a foundation for linear and COD speed [18,30], enhancements in this capacity could also translate to other traits important for football.

Strength is measured via different methods in football players, sometimes with 1RM tests [5,21,22], or maximal repetition assessments [12,28,38,39], such as that from the current study. Maximal repetition tests assess strength endurance as opposed to maximal strength. As a result, it is difficult to contextualize the data from this study with previous research. Nevertheless, when using methods to normalize the strength assessment across different positions using tonnage, and ratio-scaled and allometrically scaled tonnage, no significant differences were established between the position groups (Table 4). As previously noted, the coaches for this football squad did not test maximal strength due to a perceived risk of injury [34,35,36]. When defined by maximal strength tests, BSK and LM have been shown to be the strongest players within a team [15,16,21,22]. However, as the loads used in this study were modified by position (i.e., the SK players lifted a lighter load) [28], this essentially standardized the strength endurance data across all subjects. Maximal strength and strength endurance are important qualities for football players at all levels [15,21,22], and strength training is a clear foundation for this sport [8,57]. However, the results from this study suggest that assessing strength endurance in American football players may not provide as useful information as maximal strength data. To provide more detailed information, future research should assess the 1RM of junior college players in strength tests common to football (e.g., bench press, front and back squat, power clean), to further profile the traits of these athletes.

There are certain limitations for this study that should be acknowledged. Due to lower numbers, the offensive (centers, guards, and tackles) and defensive (defensive tackles and ends) LM were combined into one group in this study. Future investigations of junior college football players should investigate these positions separately, if the sample size is appropriate. Furthermore, the sample from this study is only representative of the school from which they were recruited; the physical performance characteristics of the current subjects may not be typical of all junior college players. The sample size for the current study also did not permit between group comparisons, due to certain positions having very small sample sizes (e.g., QB = 2 subjects, TE = 1 subject). This also meant that the numbers between the three groups in this study were not equal (i.e., SK = 31; BSK = 18; LM = 13). Indeed, the playing group from this school featured a disproportionate number of WR and DB. It would seem likely that some of these players may switch positions closer to the start of the season; however, at the time of testing these were the positions for which the players declared. It would be beneficial for further analyses of junior college football players to draw from multiple schools to increase the sample size. This study also featured only muscular endurance tests as measures of strength, due to the philosophies of the coaching staff. Future research should investigate maximal strength qualities of junior college players. No physiological measures such as heart rate or blood lactate were taken in this study. The validity of using these measures in jump, speed, and strength tests has been questioned [58,59,60], which is why there were not included in this research. Nonetheless, direct measurement of these physiological characteristics in response to typical football assessment could be an avenue for future studies. Within the context of these limitations, this is the first study to provide a comprehensive analysis of junior college football players. The results indicated that the characteristics of different position groups is typical of football players. However, the results of this study also highlighted that there are potentially certain traits (e.g., body mass, linear and COD speed, and lower-body power) that should be targeted for improvement when a junior college player transfers to a higher level of collegiate play.

## 5. Conclusions

The results indicated that junior college football players demonstrated relatively similar traits that have been shown by high school-aged [6,13,20] and collegiate [12,21,22,23] players when comparing between position groups (i.e., SK and BSK players tend to be faster and physically smaller when compared to LM). As this study has provided the most detailed investigation of junior college football players currently in the scientific literature, the data from this research could contribute to normative profiling. This data could be useful for coaches at the high school, junior college, and NCAA levels. Although coaches should directly assess players they are recruiting, comparisons to data such as that from this study can provide a greater context. The study data highlighted potential areas for improvement in junior college football players who wish to progress to Division I competition. Certain players may need to increase body mass in order to be physically competitive at the next level. All players should attempt to improve linear (both acceleration and maximal velocity running) and COD speed, in addition to lower-body power. Although improvement in these characteristics will not guarantee success at the next level [26,37], it will at least allow players to be more physically prepared for the rigors of higher competition. Finally, future research should measure maximal strength in junior college football players so that more specific recommendations can be provided with regards this capacity.

## Figures and Tables

**Table 1:** Characteristics (mean ± SD; 95% CI) by position (QB, RB, WR, TE, DB, LB, and LM) and position groupings (LM, SK (WR and DB), and BSK (QB, RB, TE, and LB)) in age, height, and body mass, and pairwise comparison data.

Position	Age (Years)		Height (m)		Body Mass (kg)	
QB (*n* = 2)	19.00 ± 0.00		1.84 ± 0.06		85.05 ± 1.61	
	(19.00–19.00)		(1.33–2.35)		(70.62–99.47)	
RB (*n* = 7)	20.00 ± 1.41		1.74 ± 0.08		92.15 ± 14.65	
	(18.69–21.31)		(1.67–1.82)		(78.60–105.69)	
WR (*n* = 13)	20.23 ± 1.17		1.85 ± 0.07		85.80 ± 5.79	
	(19.53–20.94)		(1.81–1.90)		(82.30–89.30)	
TE (*n* = 1)	19.00 ± 0.00		1.93 ± 0.00		107.96 ± 0.00	
	(–)		(–)		(–)	
DB (*n* = 18)	20.22 ± 2.18		1.79 ± 0.05		82.81 ± 6.78	
	(19.14–21.31)		(1.76–1.81)		(79.44–86.18)	
LB (*n* = 8)	20.38 ± 1.30		1.84 ± 0.04		102.68 ± 4.69	
	(19.29–21.46)		(1.80–1.87)		(98.76–106.61)	
LM (*n* = 13)	20.00 ± 1.58		1.88 ± 0.05		112.04 ± 10.26	
	(19.04–20.96)		(1.85–1.91)		(105.84–118.24)	
BSK (*n* = 18)	20.00 ± 1.28		1.81 ± 0.08 *		96.92 ± 11.56 *	
	(19.36–20.64)		(1.77–1.85)		(91.17–102.67)	
SK (*n* = 31)	20.23 ± 1.21		1.82 ± 0.07 *		84.06 ± 6.46 *^,§^	
	(19.56–20.89)		(1.79–1.84)		(81.69–86.43)	
*Pairwise Comparisons*						
	*p* value	*d*	*p* value	*d*	*p* value	*d*
SK-BSK	1.000	0.18	1.000	0.13	<0.001	1.37
SK-LM	1.000	0.16	0.030	0.90	<0.001	3.26
BSK-LM	1.000	0.00	0.019	1.05	<0.001	1.38

* Significantly (*p* < 0.05) different from the LM group; § Significantly (*p* < 0.05) different from the BSK group.

**Table 2:** Characteristics (mean ± SD; 95% CI) by position (QB, RB, WR, TE, DB, LB, and LM) and position groupings (LM, SK (WR and DB), and BSK (QB, RB, TE, and LB)) in 0–4.57 m, 0–9.14 m, 0–36.58 m, pro-agility shuttle, and three-cone drill times, and pairwise comparison data.

Position	0–4.57 m (s)		0–9.14 m (s)		0–36.58 m (s)		Pro-Agility Shuttle (s)		Three-Cone Drill (s)	
QB (*n* = 2)	1.110 ± 0.056		1.800 ± 0.057		5.105 ± 0.078		–		8.040 ± 0.000	
	(0.602–1.618)		(1.292–2.308)		(4.406–5.804)				(–)	
RB (*n* = 7)	1.070 ± 0.067		1.734 ± 0.104		4.988 ± 0.412		4.745 ± 0.357		7.566 ± 0.239	
	(0.987–1.153)		(1.605–1.863)		(4.477–5.499)		(4.177–5.313)		(7.269–7.863)	
WR (*n* = 13)	1.012 ± 0.073		1.659 ± 0081		4.811 ± 0.207		4.469 ± 0.222		7.444 ± 0.351	
	(0.960–1.064)		(1.601–1.717)		(4.663–4.959)		(4.310–4.628)		(7.120–7.769)	
TE (*n* = 1)	1.040 ± 0.000		1.680 ± 0.000		4.840 ± 0.000		4.300 ± 0.000		–	
	(–)		(–)		(–)		(–)			
DB (*n* = 18)	1.039 ± 0.088		1.703 ± 0.098		4.958 ± 0.160		4.625 ± 0.147		7.658 ± 0.235	
	(0.983–1.095)		(1.640–1.765)		(4.857–5.060)		(4.532–4.718)		(7.477–7.839)	
LB (*n* = 8)	1.000 ± 0.018		1.670 ± 0.058		4.983 ± 0.235		4.492 ± 0.076		7.598 ± 0.196	
	(0.971–1.029)		(1.578–1.762)		(4.609–5.356)		(4.398–4.586)		(7.285–7.910)	
LM (*n* = 13)	1.080 ± 0.064		1.804 ± 0.084		5.402 ± 0.266		4.970 ± 0.272		8.252 ± 0.339	
	(1.031–1.129)		(1.740–1.869)		(5.198–5.607)		(4.632–5.308)		(7.896–8.607)	
BSK (*n* = 18)	1.051 ± 0.061		1.719 ± 0.086		4.993 ± 0.286 *		4.574 ± 0.265*		7.626 ± 0.244 *	
	(1.012–1.090)		(1.664–1.774)		(4.812–5.175)		(4.384–4.763)		(7.451–7.801)	
SK (*n* = 31)	1.027 ± 0.081		1.683 ± 0.091 *		4.891 ± 0.193 *		4.554 ± 0.197*		7.564 ± 0.301*	
	(0.991–1.063)		(1.642–1.723)		(4.806–4.977)		(4.467–4.642)		(7.404–7.725)	
*Pairwise Comparisons*										
	*p* value	*d*	*p* value	*d*	*p* value	*d*	*p* value	*d*	*p* value	*d*
SK-BSK	1.000	0.33	0.773	0.41	0.713	0.42	1.000	0.09	1.000	0.23
SK-LM	0.216	0.73	0.004	1.38	<0.001	2.20	0.002	1.75	<0.001	2.15
BSK-LM	1.000	0.46	0.104	1.00	0.001	1.48	0.009	1.47	0.001	2.12

* Significantly (*p* < 0.05) different from the LM group.

**Table 3:** Characteristics (mean ± SD; 95% CI) by position (QB, RB, WR, TE, DB, LB, and LM) and position groupings (LM, SK (WR and DB), and BSK (QB, RB, TE, and LB)) in VJ height and peak power, and SBJ distance and relative SBJ, and pairwise comparison data.

Position	VJ Height (m)		VJ Peak Power (w)		SBJ (m)		Relative SBJ (m·BM^−1^)	
QB (*n* = 2)	–		–		2.55 ± 0.06		0.030 ± 0.001	
					(2.04–3.06)		(0.017–0.043)	
RB (*n* = 7)	0.62 ± 0.20		4324.81 ± 1090.29		2.50 ± 0.41		0.029 ± 0.008	
	(0.29–0.94)		(2589.91–6059.71)		(1.98–3.02)		(0.019–0.038)	
WR (*n* = 13)	0.78 ± 0.11		3787.64 ± 264.78		2.87 ± 0.20		0.033 ± 0.002	
	(0.71–0.86)		(3609.76–3965.52)		(2.75–2.99)		(0.032–0.035)	
TE (*n* = 1)	0.69 ± 0.00		4951.64 ± 0.00		2.87 ± 0.00		0.027 ± 0.000	
	(–)		(–)		(–)		(–)	
DB (*n* = 18)	0.71 ± 0.04		3645.14 ± 329.09		2.58 ± 0.15		0.031 ± 0.002	
	(0.68–0.73)		(3455.12–3835.15)		(2.49–2.66)		(0.030–0.033)	
LB (*n* = 8)	0.71 ± 0.06		4829.13 ± 126.68		2.57 ± 0.20		0.025 ± 0.002	
	(0.64–0.77)		(4696.19–4962.07)		(2.39–2.76)		(0.023–0.027)	
LM (*n* = 13)	0.58 ± 0.07		5273.19 ± 684.35		2.35 ± 0.28		0.021 ± 0.004	
	(0.52–0.63)		(4783.63–5762.74)		(2.18–2.52)		(0.019–0.024)	
BSK (*n* = 18)	0.67 ± 0.13		4656.88 ± 659.73 *		2.56 ± 0.27		0.027 ± 0.005 *	
	(0.59–0.76)		(4231.67–5100.09)		(2.41–2.72)		(0.024–0.030)	
SK (*n* = 31)	0.74 ± 0.09 *		3707.84 ± 305.10 *^,§^		2.72 ± 0.23 *		0.032 ± 0.003 *^,§^	
	(0.71–0.78)		(3581.90–3833.78)		(2.63–2.81)		(0.031–0.033)	
*Pairwise Comparisons*									
	*p* value	*d*	*p* value	*d*	*p* value	*d*	*p* value	*d*
SK-BSK	0.148	0.63	<0.001	1.85	0.203	0.64	<0.001	1.21
SK-LM	<0.001	1.98	<0.001	2.95	<0.001	1.44	<0.001	3.11
BSK-LM	0.091	0.86	0.022	0.92	0.086	0.76	<0.001	1.33

* Significantly (*p* < 0.05) different from the LM group; § Significantly (*p* < 0.05) different from the BSK group.

**Table 4:** Characteristics (mean ± SD; 95% CI) by position (QB, RB, WR, TE, DB, LB, and LM) and position groupings (LM, SK (WR and DB), and BSK (QB, RB, TE, and LB)) in bench press repetition number, tonnage, and ratio- scaled and allometrically scaled tonnage.

Position	Repetition Number		Tonnage (kg)		Ratio-Scaled Tonnage (kg·BM^−1^)		Allometrically Scaled Tonnage (kg·BM^−0.67^)	
QB (*n* = 2)	2.00 ± 0.00		204.00 ± 0.00		2.37 ± 0.00		3.53 ± 0.00	
	(–)		(–)		(–)		(–)	
RB (*n* = 7)	11.17 ± 4.88		1085.00 ± 417.65		11.96 ± 5.43		17.85 ± 8.11	
	(6.05–16.28)		(646.70–1523.30)		(6.26–17.66)		(9.34–26.36)	
WR (*n* = 13)	10.00 ± 3.27		840.00 ± 274.34		9.69 ± 2.70		14.46 ± 4.03	
	(8.03–11.97)		(674.22–1005.78)		(8.06–11.32)		(12.03–16.89)	
TE (*n* = 1)	12.00 ± 0.00		1224.00 ± 0.00		11.34 ± 0.00		16.92 ± 0.00	
	(–)		(–)		(–)		(–)	
DB (*n* = 18)	11.69 ± 6.19		981.75 ± 520.27		11.66 ± 5.68		17.40 ± 8.48	
	(8.39–14.99)		(704.52–1258.98)		(8.63–14.68)		(12.88–21.91)	
LB (*n* = 8)	13.25 ± 7.05		1351.50 ± 718.67		13.06 ± 6.64		19.49 ± 9.91	
	(7.36–19.14)		(750.68–1952.32)		(7.51–18.61)		(11.21–27.78)	
LM (*n* = 13)	10.64 ± 5.64		1028.50 ± 582.64		8.99 ± 4.86		13.41 ± 7.26	
	(6.84–14.43)		(658.31–1398.69)		(5.90–12.08)		(8.80–18.02)	
BSK (*n* = 18)	11.69 ± 6.23		1171.88 ± 618.09		11.87 ± 6.10		17.72 ± 9.10	
	(8.37–15.01)		(842.52–1501.23)		(8.62–15.12)		(12.87–22.57)	
SK (*n* = 31)	10.93 ± 5.09		918.21 ± 427.10		10.77 ± 4.62		16.08 ± 6.90	
	(9.00–12.87)		(755.45–1080.67)		(9.02–12.53)		(13.46–18.71)	
*Pairwise Comparisons*								
	*p* value	*d*	*p* value	*d*	*p* value	*d*	*p* value	*d*
SK-BSK	1.000	0.13	0.368	0.48	1.000	0.20	1.000	0.20
SK-LM	1.000	0.05	1.000	0.22	0.941	0.38	0.942	0.38
BSK-LM	1.000	0.18	1.000	0.24	0.438	0.52	0.439	0.52

**Table 5:** Characteristics (mean ± SD; 95% CI) by position (QB, RB, WR, TE, DB, LB, and LM) and position groupings (LM, SK (WR and DB), and BSK (QB, RB, TE, and LB)) in front squat repetition number, tonnage, and ratio and allometrically scaled tonnage.

Position	Repetition Number		Tonnage (kg)		Ratio-Scaled Tonnage (kg·BM^−1^)		Allometrically Scaled Tonnage (kg·BM^−0.33^)	
QB (*n* = 2)	4.00 ± 0.00		408.00 ± 0.00		4.73 ± 0.00		14.35 ± 0.00	
	(–)		(–)		(–)		(–)	
RB (*n* = 7)	11.83 ± 7.00		1141.00 ± 609.36		12.90 ± 7.46		39.09 ± 22.60	
	(4.49–19.18)		(501.51–1780.49)		(5.07–20.73)		(15.38–62.81)	
WR (*n* = 13)	14.55 ± 6.65		1221.82 ± 558.92		14.30 ± 6.14		43.34 ± 18.61	
	(10.08–19.02)		(846.33–1597.30)		(10.18–18.43)		(30.84–55.85)	
TE (*n* = 1)	19.00 ± 0.00		1938.00 ± 0.00		17.95 ± 0.00		54.40 ± 0.00	
	(–)		(–)		(–)		(–)	
DB (*n* = 18)	14.40 ± 9.05		1209.60 ± 759.83		13.81 ± 7.72		41.84 ± 23.40	
	(7.93–20.87)		(66.05–1753.15)		(8.28–19.33)		(25.11–58.58)	
LB (*n* = 8)	19.00 ± 7.77		1938.00 ± 792.72		18.57 ± 7.32		56.27 ± 22.18	
	(10.84–27.16)		(1106.09–2769.91)		(10.89–26.25)		(32.99–79.54)	
LM (*n* = 13)	9.75 ± 3.88		994.50 ± 395.98		8.73 ± 3.19		26.46 ± 9.67	
	(6.50–13.00)		(663.45–1325.55)		(6.06–11.40)		(18.37–34.55)	
BSK (*n* = 18)	14.86 ± 8.04		1487.14 ± 799.28		15.11 ± 7.66		45.78 ± 23.21	
	(10.22–19.50)		(1025.65–1948.63)		(10.68–19.53)		(32.38–59.18)	
SK (*n* = 31)	14.48 ± 7.68		1216.00 ± 645.01		14.07 ± 6.76		42.63 ± 20.50	
	(10.98–17.97)		(922.40–1509.60)		(10.99–17.15)		(33.30–51.96)	
*Pairwise Comparisons*								
	*p* value	*d*	*p* value	*d*	*p* value	*d*	*p* value	*d*
SK-BSK	1.000	0.05	0.734	0.37	1.000	0.14	1.000	0.14
SK-LM	0.379	0.81	1.000	0.41	0.178	1.01	0.178	1.01
BSK-LM	0.365	0.78	0.308	0.78	0.107	1.09	0.107	1.09

## Abbreviations

The following abbreviations are used in this manuscript:

ANOVA	Analysis of variance
NCAA	National Collegiate Athletic Association
QB	Quarterbacks
RB	Running backs
WR	Wide receivers
TE	Tight ends
DB	Defensive backs
LB	Linebackers
LM	Linemen
BSK	Big skill
SK	Skill
1RM	One repetition-maximum
VJ	Vertical jump
kg	Kilograms
COD	Change-of-direction
m	Meter
SBJ	Standing broad jump
ICC	Intra-class correlation coefficient
CV	Coefficient of variation
w	Watts
m·kg^−1^	Meters per kilogram
kg·BM^−1^	Kilograms lifted per kilogram body mass
kg·BM^−0.67^	Kilograms lifted per kilogram body mass with the allometric scaling factor for the bench press
kg·BM^−0.33^	Kilograms lifted per kilogram body mass with the allometric scaling factor for the front squat
s	Seconds
SD	Standard deviation
CI	Confidence intervals
*p*	Significance
*d*	Effect size

## References

[1] Roy D.P., Graeff T.R., Harmon S.K. (2008). Repositioning a university through NCAA Division I-A football membership. J. Sport Manag..

[2] Amorose A.J., Horn T.S. (2000). Intrinsic motivation: Relationships with collegiate athletes’ gender, scholarship status, and perceptions of their coaches’ behavior. J. Sport Exerc. Psychol..

[3] Langelett G. (2003). The relationship between recruiting and team performance in Division 1A college football. J. Sports Econ..

[4] Bergman S.A., Logan T.D. (2008). The effect of recruit quality on college football team performance. J. Sports Econom..

[5] Carbuhn A.F., Womack J.W., Green J.S., Morgan K., Miller G.S., Crouse S.F. (2014). Performance and blood pressure characteristics of first-year national collegiate athletic association Division I football players. J. Strength Cond. Res..

[6] Dupler T.L., Amonette W.E., Coleman A.E., Hoffman J.R., Wenzel T. (2010). Anthropometric and performance differences among high-school football players. J. Strength Cond. Res..

[7] Kaiser G.E., Womack J.W., Green J.S., Pollard B., Miller G.S., Crouse S.F. (2008). Morphological profiles for first-year National Collegiate Athletic Association Division I football players. J. Strength Cond. Res..

[8] Hoffman J.R., Ratamess N.A., Kang J. (2011). Performance changes during a college playing career in NCAA Division III football athletes. J. Strength Cond. Res..

[9] Stodden D.F., Galitski H.M. (2010). Longitudinal effects of a collegiate strength and conditioning program in American football. J. Strength Cond. Res..

[10] Dos Remedios K.A., dos Remedios R.L., Loy S.F., Holland G.J., Vincent W.J., Conley L.M., Heng M. (1995). Physiological and field test performance changes of community college football players over a season. J. Strength Cond. Res..

[11] Dos Remedios R., Holland G. (1992). Physical and performance characteristics of community college football players. Strength Cond. J..

[12] Sierer S.P., Battaglini C.L., Mihalik J.P., Shields E.W., Tomasini N.T. (2008). The National Football League Combine: Performance differences between drafted and nondrafted players entering the 2004 and 2005 drafts. J. Strength Cond. Res..

[13] Ghigiarelli J.J. (2011). Combine performance descriptors and predictors of recruit ranking for the top high school football recruits from 2001 to 2009: Differences between position groups. J. Strength Cond. Res..

[14] Hoffman J.R. (2008). The applied physiology of American football. Int. J. Sports Physiol. Perform..

[15] Pincivero D.M., Bompa T.O. (1997). A physiological review of American football. Sports Med..

[16] Berg K., Latin R.W., Baechle T. (1990). Physical and performance characteristics of NCAA Division I football players. Res. Q. Exerc. Sport..

[17] Kraemer W.J., Torine J.C., Silvestre R., French D.N., Ratamess N.A., Spiering B.A., Hatfield D.L., Vingren J.L., Volek J.S. (2005). Body size and composition of National Football League players. J. Strength Cond. Res..

[18] Lockie R.G., Jeffriess M.D., Schultz A.B., Callaghan S.J. (2012). Relationship between absolute and relative power with linear and change-of-direction speed in junior American football players from Australia. J. Aust. Strength Cond..

[19] Jacobson B.H. (2012). Anthropometric cross-sectional comparisons of college football players and potential health implications. J. Strength Cond. Res..

[20] Lockie R.G., Schultz A.B., Callaghan S.J., Jeffriess M.D. (2012). Physiological profile of national-level junior American football players in Australia. Serbian J. Sports Sci..

[21] Garstecki M.A., Latin R.W., Cuppett M.M. (2004). Comparison of selected physical fitness and performance variables between NCAA Division I and II football players. J. Strength Cond. Res..

[22] Secora C.A., Latin R.W., Berg K.E., Noble J.M. (2004). Comparison of physical and performance characteristics of NCAA Division I football players: 1987 and 2000. J. Strength Cond. Res..

[23] Stuempfle K.J., Katch F.I., Petrie D.F. (2003). Body composition relates poorly to performance tests in NCAA Division III football players. J. Strength Cond. Res..

[24] Schmidt W.D. (1999). Strength and physiological characteristics of NCAA Division III American football players. J. Strength Cond. Res..

[25] Berg K., Latin R.W. (1995). Comparison of physical and performance characteristics of NCAA Division I basketball and football players. J. Strength Cond. Res..

[26] Robbins D.W. (2010). The National Football League (NFL) combine: Does normalized data better predict performance in the NFL draft?. J. Strength Cond. Res..

[27] McGee K.J., Burkett L.N. (2003). The National Football League combine: A reliable predictor of draft status?. J. Strength Cond. Res..

[28] Brechue W.F., Mayhew J.L. (2009). Upper-body work capacity and 1RM prediction are unaltered by increasing muscular strength in college football players. J. Strength Cond. Res..

[29] Winter E.M., Maughan R.J. (2009). Requirements for ethics approvals. J. Sports Sci..

[30] Lockie R.G., Murphy A.J., Knight T.J., de Janse Jonge X.A.K. (2011). Factors that differentiate acceleration ability in field sport athletes. J. Strength Cond. Res..

[31] Nuzzo J.L., Anning J.H., Scharfenberg J.M. (2011). The reliability of three devices used for measuring vertical jump height. J. Strength Cond. Res..

[32] Johnson D.L., Bahamonde R. (1996). Power output estimate in university athletes. J. Strength Cond. Res..

[33] Markovic G., Dizdar D., Jukic I., Cardinale M. (2004). Reliability and factorial validity of squat and countermovement jump tests. J. Strength Cond. Res..

[34] Bilsborough J.C., Greenway K.G., Opar D.A., Livingstone S.G., Cordy J.T., Bird S.R., Coutts A.J. (2015). Comparison of anthropometry, upper-body strength, and lower-body power characteristics in different levels of Australian football players. J. Strength Cond. Res..

[35] West D.J., Owen N.J., Jones M.R., Bracken R.M., Cook C.J., Cunningham D.J., Shearer D.A., Finn C.V., Newton R.U., Crewther B.T. (2011). Relationships between force-time characteristics of the isometric midthigh pull and dynamic performance in professional rugby league players. J. Strength Cond. Res..

[36] Coutts A.J., Murphy A.J., Dascombe B.J. (2004). Effect of direct supervision of a strength coach on measures of muscular strength and power in young rugby league players. J. Strength Cond. Res..

[37] Kuzmits F.E., Adams A.J. (2008). The NFL combine: Does it predict performance in the National Football League?. J. Strength Cond. Res..

[38] Mayhew J.L., Ware J.S., Bemben M.G., Wilt B., Ward T.E., Farris B., Juraszek J., Slovak J.P. (1999). The NFL-225 test as a measure of bench press strength in college football players. J. Strength Cond. Res..

[39] Chapman P.P., Whitehead J.R., Binkert R.H. (1998). The 225–1b reps-to-fatigue test as a submaximal estimate of 1-RM bench press performance in college football players. J. Strength Cond. Res..

[40] Mann J.B., Ivey P.J., Brechue W.F., Mayhew J.L. (2014). Reliability and smallest worthwhile difference of the NFL-225 test in NCAA Division I football players. J. Strength Cond. Res..

[41] Pereira M.I.R., Gomes P.S.C. (2003). Muscular strength and endurance tests: Reliability and prediction of one repetition maximum—Review and new evidences. Rev. Bras. Med. Esporte.

[42] Gullett J.C., Tillman M.D., Gutierrez G.M., Chow J.W. (2009). A biomechanical comparison of back and front squats in healthy trained individuals. J. Strength Cond. Res..

[43] Hiscock D.J., Dawson B., Peeling P. (2015). Perceived exertion responses to changing resistance training programming variables. J. Strength Cond. Res..

[44] Jacobson B.H., Thompson B.J., Conchola E.C., Glass R. (2013). A comparison of absolute, ratio and allometric scaling methods for normalizing strength in elite American football players. J. Athl. Enhanc..

[45] Thompson B.J., Smith D.B., Jacobson B.H., Fiddler R.E., Warren A.J., Long B.C., O’Brien M.S., Everett K.L., Glass R.G., Ryan E.D. (2010). The influence of ratio and allometric scaling procedures for normalizing upper body power output in division I collegiate football players. J. Strength Cond. Res..

[46] Jaric S., Mirkov D., Markovic G. (2005). Normalizing physical performance tests for body size: A proposal for standardization. J. Strength Cond. Res..

[47] Lockie R.G., Schultz A.B., Callaghan S.J., Jeffriess M.D., Berry S.P. (2013). Reliability and validity of a new test of change-of-direction speed for field-based sports: The Change-of-Direction and Acceleration Test (CODAT). J. Sports Sci. Med..

[48] Stewart P.F., Turner A.N., Miller S.C. (2014). Reliability, factorial validity, and interrelationships of five commonly used change of direction speed tests. Scand. J. Med. Sci. Sports.

[49] Cohen J. (1988). Statistical Power Analysis for the Behavioral Sciences.

[50] Hopkins W.G. (2004). How to interpret changes in an athletic performance test. Sportscience.

[51] Kraemer W.J., Spiering B.A., Volek J.S., Martin G.J., Howard R.L., Ratamess N.A., Hatfield D.L., Vingren J.L., Ho J.Y., Fragala M.S. (2009). Recovery from a National Collegiate Athletic Association Division I football game: Muscle damage and hormonal status. J. Strength Cond. Res..

[52] Rumpf M.C., Lockie R.G., Cronin J.B., Jalilvand F. (2016). The effect of different sprint training methods on sprint performance over various distances: A brief review. J. Strength Cond. Res..

[53] Seitz L.B., Reyes A., Tran T.T., Saez de Villarreal E., Haff G.G. (2014). Increases in lower-body strength transfer positively to sprint performance: A systematic review with meta-analysis. Sports Med..

[54] Lockie R.G., Murphy A.J., Schultz A.B., Knight T.J., Janse de Jonge X.A.K. (2012). The effects of different speed training protocols on sprint acceleration kinematics and muscle strength and power in field sport athletes. J. Strength Cond. Res..

[55] De Villarreal E.S., Kellis E., Kraemer W.J., Izquierdo M. (2009). Determining variables of plyometric training for improving vertical jump height performance: A meta-analysis. J. Strength Cond. Res..

[56] De Villarreal E.S., Requena B., Cronin J.B. (2012). The effects of plyometric training on sprint performance: A meta-analysis. J. Strength Cond. Res..

[57] Jacobson B.H., Conchola E.G., Glass R.G., Thompson B.J. (2013). Longitudinal morphological and performance profiles for American, NCAA Division I football players. J. Strength Cond. Res..

[58] Egan A.D., Winchester J.B., Foster C., McGuigan M.R. (2006). Using session RPE to monitor different methods of resistance exercise. J. Sports Sci. Med..

[59] Impellizzeri F.M., Rampinini E., Coutts A.J., Sassi A., Marcora S.M. (2004). Use of RPE-based training load in soccer. Med. Sci. Sports Exerc..

[60] Lockie R.G., Murphy A.J., Scott B.R., de Janse Jonge X.A.K. (2012). Quantifying session ratings of perceived exertion for field-based speed training methods in team sport athletes. J. Strength Cond. Res..

